# SARS-CoV-2 Infection of Human Neurons Is TMPRSS2 Independent, Requires Endosomal Cell Entry, and Can Be Blocked by Inhibitors of Host Phosphoinositol-5 Kinase

**DOI:** 10.1128/jvi.00144-23

**Published:** 2023-04-11

**Authors:** Pinja Kettunen, Angelina Lesnikova, Noora Räsänen, Ravi Ojha, Leena Palmunen, Markku Laakso, Šárka Lehtonen, Johanna Kuusisto, Olli Pietiläinen, Saber H. Saber, Merja Joensuu, Olli P. Vapalahti, Jari Koistinaho, Taisia Rolova, Giuseppe Balistreri

**Affiliations:** a Neuroscience Center, HiLIFE, University of Helsinki, Helsinki, Finland; b Department of Virology, Faculty of Medicine, University of Helsinki, Helsinki, Finland; c Institute of Clinical Medicine, University of Eastern Finland, Kuopio, Finland; d A. I. Virtanen Institute for Molecular Sciences, University of Eastern Finland, Kuopio, Finland; e Department of Medicine and Clinical Research, Kuopio University Hospital, Kuopio, Finland; f Australian Institute for Bioengineering and Nanotechnology, The University of Queensland, Brisbane, Queensland, Australia; g Queensland Brain Institute, The University of Queensland, Brisbane, Queensland, Australia; Loyola University Chicago Health Sciences Campus

**Keywords:** PIK5, SARS-CoV-2, apilimod, astrocyte, brain, central nervous system infections, COVID-19, iPSC, long COVID, neuron

## Abstract

2019 coronavirus disease (COVID-19) is a disease caused by severe acute respiratory syndrome coronavirus 2 (SARS-CoV-2). In addition to respiratory illness, COVID-19 patients exhibit neurological symptoms lasting from weeks to months (long COVID). It is unclear whether these neurological manifestations are due to an infection of brain cells. We found that a small fraction of human induced pluripotent stem cell (iPSC)-derived neurons, but not astrocytes, were naturally susceptible to SARS-CoV-2. Based on the inhibitory effect of blocking antibodies, the infection seemed to depend on the receptor angiotensin-converting enzyme 2 (ACE2), despite very low levels of its expression in neurons. The presence of double-stranded RNA in the cytoplasm (the hallmark of viral replication), abundant synthesis of viral late genes localized throughout infected cells, and an increase in the level of viral RNA in the culture medium (viral release) within the first 48 h of infection suggested that the infection was productive. Productive entry of SARS-CoV-2 requires the fusion of the viral and cellular membranes, which results in the delivery of the viral genome into the cytoplasm of the target cell. The fusion is triggered by proteolytic cleavage of the viral surface spike protein, which can occur at the plasma membrane or from endosomes or lysosomes. We found that SARS-CoV-2 infection of human neurons was insensitive to nafamostat and camostat, which inhibit cellular serine proteases, including transmembrane serine protease 2 (TMPRSS2). Inhibition of cathepsin L also did not significantly block infection. In contrast, the neuronal infection was blocked by apilimod, an inhibitor of phosphatidyl-inositol 5 kinase (PIK5K), which regulates early to late endosome maturation.

**IMPORTANCE** COVID-19 is a disease caused by the coronavirus SARS-CoV-2. Millions of patients display neurological symptoms, including headache, impairment of memory, seizures, and encephalopathy, as well as anatomical abnormalities, such as changes in brain morphology. SARS-CoV-2 infection of the human brain has been documented, but it is unclear whether the observed neurological symptoms are linked to direct brain infection. The mechanism of virus entry into neurons has also not been characterized. Here, we investigated SARS-CoV-2 infection by using a human iPSC-derived neural cell model and found that a small fraction of cortical-like neurons was naturally susceptible to infection. The productive infection was ACE2 dependent and TMPRSS2 independent. We also found that the virus used the late endosomal and lysosomal pathway for cell entry and that the infection could be blocked by apilimod, an inhibitor of cellular PIK5K.

## INTRODUCTION

A variety of neurological symptoms have been observed in millions of 2019 coronavirus disease (COVID-19) patients, which has led to a hypothesis that severe acute respiratory syndrome coronavirus 2 (SARS-CoV-2) can infect brain cells. Such symptoms include fatigue, headache, impairment of concentration and memory (“brain fog”), seizures, and encephalopathy (1). Structural changes in the brain anatomy have also been observed. A magnetic resonance imaging study of 785 participants found reductions in gray matter thickness and global brain volume in combination with changes in tissue contrast and tissue damage markers in certain brain areas (2). Post mortem analysis of deceased COVID-19 patients has indicated presence of viral components in neurons, glial, and endothelial cells in different regions of the brain, including the olfactory bulb, which connects the olfactory sensory neurons of the nasal epithelium to the central nervous system via a dense network of nerves (3–9). In *in vitro* cell culture models, SARS-CoV-2 can infect neurons derived from human embryonic stem cells (ESCs) and induced pluripotent stem cell (iPSCs) in both two-dimensional (2D; monolayers) and 3D models (e.g., organoids) (7, 10–16). Some studies also reported infection in iPSC- or ESC-derived astrocytes (8, 14, 16).

Bona fide neurotropic viruses, such as rabies virus, poliovirus, or tick-borne encephalitis virus, cause severe neuronal infection that spreads to large areas of the brain with paralyzing or lethal consequences (17–20). The potential of SARS-CoV-2 to infect very limited areas of the human brain, and the possibility that this nonlethal infection could be transient, could explain some of the neurological manifestations in patients that suffer long COVID.

How SARS-CoV-2 enters brain cells is not clear. Also, whether the virus can spread from the initially infected neurons is debated, with studies showing both productive (11, 15) and nonproductive infection (10, 13, 21).

Analysis of the SARS-CoV-2 infection in cell lines and primary respiratory epithelial cell models has indicated that the virus has at least two possible entry routes: (i) endocytosis and fusion from lysosomes or (ii) direct fusion at the plasma membrane (22–24). Both mechanisms require a mildly acidic environment (pH of <6.8) and the activation of the viral surface protein spike (S) by cellular proteases such as cathepsin-L (in lysosomes) or serine proteases such as transmembrane serine protease 2 (TMPRSS2; at the plasma membrane). Depending on the cellular availability of these proteases, infection can occur within lysosomes or at the cell surface. Inhibitors of endosome maturation (e.g., phosphatidyl-inositol 5 kinase [PIK5K] inhibitors) block virus infection from endosomes or lysosomes (22). Inhibitors of serine proteases (e.g., nafamostat and camostat) block infection from the plasma membrane. Here, we used authentic SARS-CoV-2 to investigate the infection route and the spreading potential of the virus in 2D-cultured human iPSC-derived neurons, astrocytes, and neuron-astrocyte cocultures.

## RESULTS AND DISCUSSION

### Characterization of human iPSC-derived neurons and astrocytes.

To study the viral entry mechanisms in human brain cells, we set up a human iPSC-derived neuron-astrocyte coculture system in a 96-well plate format. The iPSC lines used for neuron and astrocyte differentiation are listed in Table 1. All lines had the normal karyotypes and they expressed known pluripotency markers podocalyxin (TRA-1-81), stage-specific embryonic antigen 4 (SSEA-4), octamer-binding transcription factor (Oct)-4, and homeobox transcription factor Nanog at the protein level and *POU5F1* (Oct-3/4), *NANOG*, and sex determining region Y box 2 (*SOX2*) at the mRNA level (see Fig. S1 in the supplemental material). Since the iPSC lines used in this study were reprogrammed using a Sendai virus-based vector, we also determined that residual expression of Sendai virus RNA was below the detection limit.

**TABLE 1:** List of iPSC lines used

ID	Gender	Donor age (yrs)	Cell type	Corresponding figure(s)	Previously published in reference
MADGIC 1cl7	Male	67	Astrocyte	1B, C, and G, 2A, B, and D to H, 3A and B	45
MADGIC 4cl1	Male	65	Astrocyte, neuron	1A to E and G, 3E and F	45
MADGIC 6cl1	Male	63	Neuron	1A, D, and G, 2A, B, D to F, I, and J, 3A and B	46
MADGIC 7cl1	Male	66	Neuron	1A, D, and E	Previously unpublished
MADGIC 8cl1	Male	64	Astrocyte, neuron	1, 2, 3A, B, and G, 4	46
MADGIC 9cl1	Male	58	Neuron	1A, D, and E, 3E and F	Previously unpublished
MADGIC 12cl2	Male	58	Astrocyte, neuron	1, 2C, 3E and F	47

**FIG 1:**
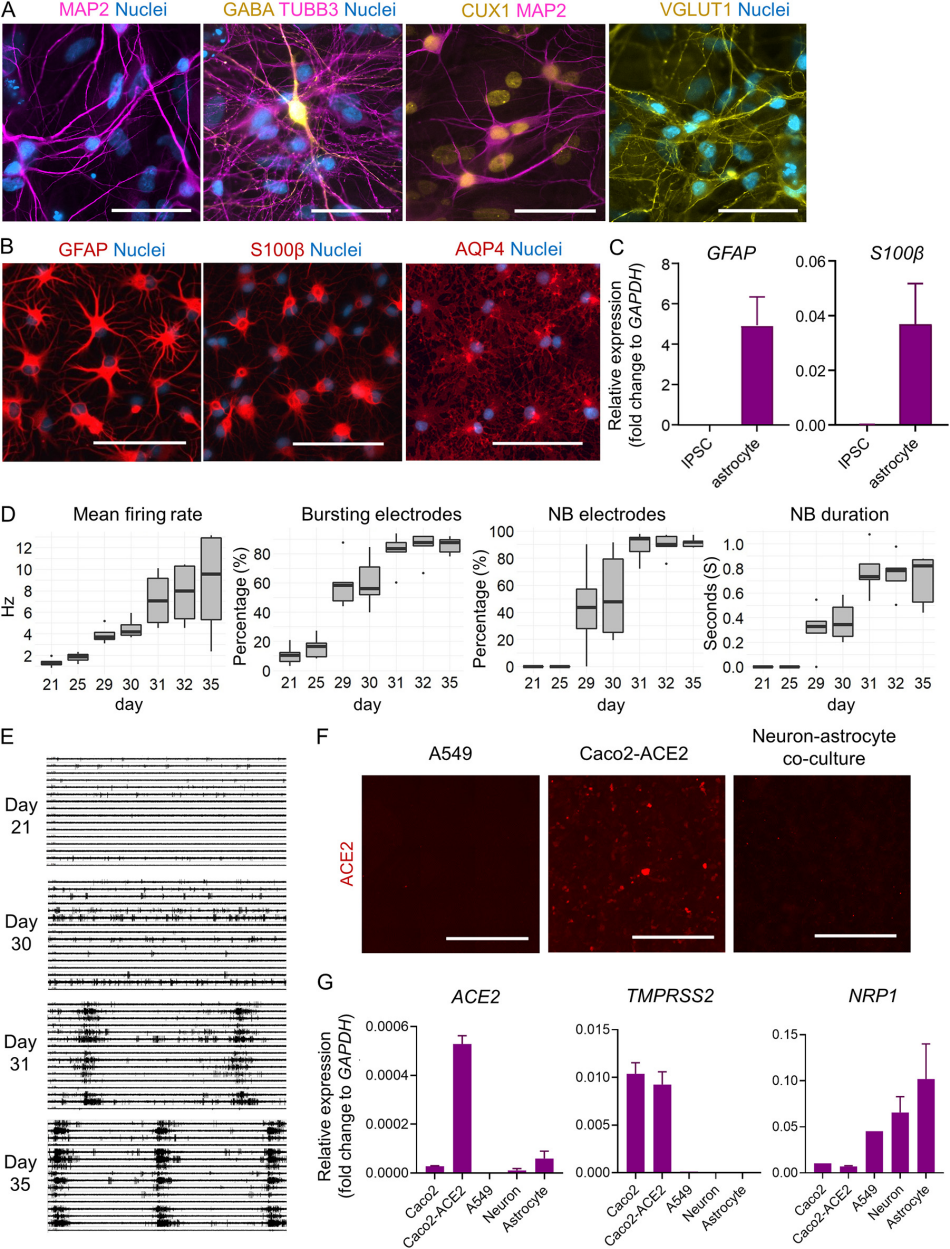
Characterization of iPSC-derived neurons and astrocytes. (A) Representative images of immunocytochemical staining of iPSC-derived NGN2-neurons with MAP2, TUBB3, GABA, CUX1, and VGLUT1 antibodies. Scale bar, 50 μm. (B) Representative images of immunocytochemical staining of iPSC-derived astrocytes with GFAP, S100β, and AQP4 antibodies. Scale bar, 100 μm. (C) Mean normalized mRNA expression of astrocyte markers *GFAP* and *S100β* in iPSCs and iPSC-derived astrocytes. Values are normalized to human *GAPDH* expression. *N* = 4 cell lines. (D) Quantification of the mean firing rate (in hertz), percentage of electrodes partaking in bursts, percentage of electrodes partaking in network bursts, and network burst duration (in seconds) in iPCS-derived neuron-astrocyte cocultures grown for the indicated times. *N* = 3 cell lines. (E) Representative images of MEA recordings from neuron-astrocyte cocultures at days 21, 30, 31, and 35. (F) Representative fluorescence images of indicated cell lines after immunocytochemical staining using antibodies against ACE2. Scale bar, 1,000 μm. (G) Mean normalized mRNA expression of *ACE2*, *TMPRSS2*, and *NRP1* in Caco2, Caco2-ACE2, A549, neuron, and astrocyte cell lines. Values were normalized to human *GAPDH* expression. *N* = 2 to 4 cell lines.

We used 6 different iPSC lines (Table 1) to obtain cortical-like neurons using the overexpression of tetracycline-inducible neurogenin 2 (NGN2) in combination with dual SMAD/WNT inhibition. We confirmed the neuronal identity of the iPSC-derived cells by positive staining for the neuronal markers microtubule-associated protein 2 (MAP2) and class III β-tubulin (TUBB3) (Fig. 1A). The majority of the neurons displayed nuclear expression of Cut-like homeobox 1 (CUX1), a marker for upper cortical layer neurons (layers II and III) (Fig. 1A) and lacked COUP-TF-interacting protein 2 (CTIP2), which is a marker for lower cortical layers V and VI (data not shown). In addition, the majority of the neurons showed robust staining with vesicular glutamate transporter 1 (VGLUT1) (Fig. 1A). Together, these data implied that our cultures contained mainly excitatory glutamatergic neurons of the upper cortical layer (II and III) identity. However, some neurons displayed positive staining for gamma-aminobutyric acid (GABA) (Fig. 1A), which suggested that the cultures also contained small subsets of inhibitory GABAergic interneurons.

We also generated astrocytes from 4 different iPSC lines using the sphere-based protocol described in Materials and Methods. The identity of iPSC-derived astrocytes was confirmed by staining with the astrocyte markers glial fibrillary acidic protein (GFAP), S100 calcium binding protein β (S100β), and aquaporin 4 (AQP4) (Fig. 1B). The expression of *GFAP* and *S100β* mRNAs in the iPSC-derived human astrocytes was further confirmed by reverse transcription-quantitative PCR (qRT-PCR), and neither of these was detected in the parental iPSCs (Fig. 1C).

To create a more complex model for studying SARS-CoV-2 infection, we mixed iPSC-derived neurons and astrocytes at a 1:1 ratio and cultured them together for several weeks. To evaluate the maturity and functionality of the neuron-astrocyte cocultures, we used a 24-well microelectrode array (MEA) system. The ability of neurons to form connected, electrically active networks is an important hallmark of their functionality.

In MEA experiments, bursts are periods of increased electrical activity recorded from a group of neurons connected to a recording electrode (single-electrode bursting). During network bursting, multiple electrodes record increased activity at the same time, which indicates that there is connectivity between different parts of the neuronal network. The cocultures used in this study started developing electric bursting activity after 3 weeks of maturation, as seen by a gradual increase in mean firing rate and the percentage of bursting electrodes (Fig. 1D). Network maturation in our neuron-astrocyte cocultures reached a plateau by day 31, when most of the recording electrodes (>80%) recorded both single-electrode bursting and network bursting and the network burst duration had reached a duration of 0.7s (Fig. 1D). Representative images of MEA recordings from all 16 electrodes at days 21, 30, 31, and 35 are shown in Fig. 1E (10-s interval). Since our neuron-astrocyte cocultures formed a connected, electrically active network as expected of functional neurons by days 30 to 32, we chose this time point for SARS-CoV-2 infection experiments.

Finally, we used qRT-PCR to assess the expression of cell surface molecules known to be important for SARS-CoV-2 infection in the respiratory tract. The level of the main entry receptor ACE2 in iPSC-derived neurons was below immunofluorescence detection (Fig. 1F). At the mRNA level, *ACE2* expression was detectable in both iPSC-derived neurons and astrocytes. However, the levels were low compared to those for the Caco2-ACE2 cell line, which overexpresses ACE2 receptor (Fig. 1G and Fig. S2). The level of *TMPRSS2* mRNA expression in iPSC-derived neurons and astrocytes was at the same level with the negative control cell line A549, which does not express this gene (Fig. 1G and Fig. S2). Both neurons and astrocytes expressed detectable levels of the entry cofactor *neuropilin 1* (*NRP1*) mRNA (Fig. 1G and Fig. S2). Neuropilin is a protein that controls axonal development in neurons and has recently been shown to facilitate SARS-CoV-2 infection in ACE2-expressing cells (25, 26).

In summary, the neuron-astrocyte coculture model displayed electric activity typical of mature neurons, with correctly expressed cell markers. At the RNA level, both neurons and astrocytes endogenously expressed *NRP1* and low amounts of *ACE2*, but not *TMPRSS2*.

### SARS-CoV-2 selectively infects neurons in an iPSC-derived neuron-astrocyte coculture model, and the infection appears productive.

**FIG 2:**
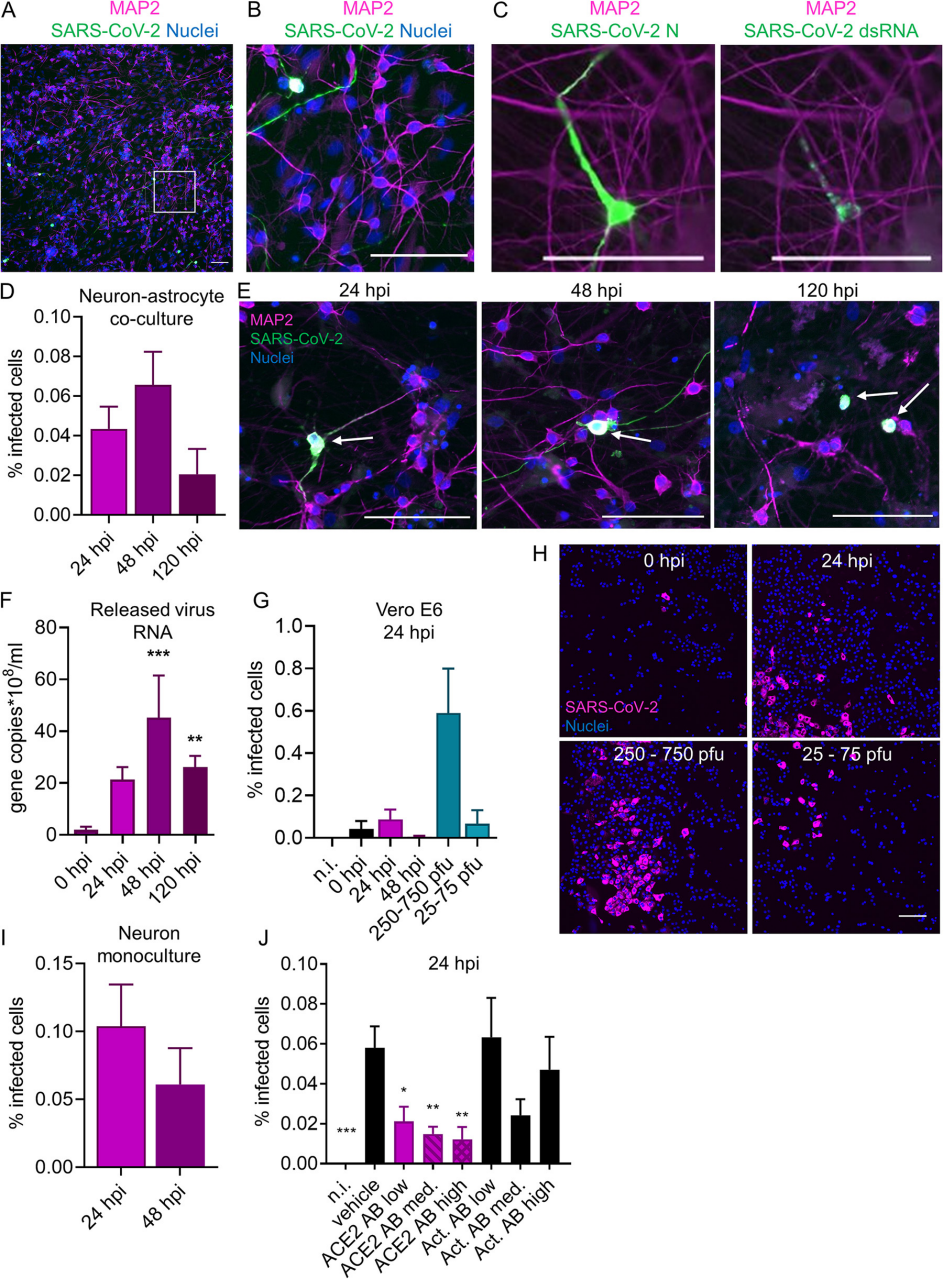
Infection of iPSC-derived neural cultures by SARS-CoV-2 is mainly dependent on the ACE2 receptor and does not spread efficiently. (A to C) Representative images of hiPCS-derived neuron-astrocyte cocultures infected with SARS-CoV-2 Wuhan virus for 48 h. Infected cells were identified by immunofluorescence detection (green) of the viral protein N (A and B) or N and double-stranded RNA colocalizing in the same infected cell (C). The image in panel B represents an enlarged area from the white boxed area in panel A. Nuclei were visualized with Hoechst DNA staining and neurons by immunodetection on MAP2 protein (magenta). (D) Percentage of SARS-COV-2-infected neurons in neuron-astrocyte cocultures at 24, 48, and 120 hpi. Neurons were identified by automated classification based on the fluorescence of MAP2. Astrocytes, identified as MAP2-negative cells, were never found infected. *N* = 6 wells per treatment. For each group, more than 10,000 cells were imaged and analyzed. (E) Representative fluorescence images of infected neuron-astrocyte cocultures from the experiment shown in panel D, at the indicated time points. SARS-CoV-2-infected cells (green, indicated by white arrows) identified by immunodetection of viral N protein, neurons by MAP2 (magenta), and nuclei (blue) by Hoechst DNA staining. (F) qRT-PCR of the extracellular medium withdrawn at the indicated time points after infection of neuron-astrocyte cocultures, using primers against SARS-COV-2 genome. Values are expressed as the number of genome copies per milliliter by comparison with a standard curve using known amounts of viral RNA. *N* = 6 wells per group. Significance was tested between 0 hpi and 24 hpi or 48 hpi (not significant [n.s.]). (G) Infection of Vero E6 cells with medium collected from infected neuron-astrocyte cocultures at 0, 24, or 48 hpi, or with the indicated amounts of a virus stock previously produced in Vero E6-TMPRSS2 cells (250 to 750 PFU or 25 to 75 PFU were used to infect each well of 96-well plates). *N* = 6 wells per group. Significance is shown in comparison to 0 hpi. (H) Representative immunofluorescent images of VERO E6 cells infected as described for panel G. Nuclei (blue) were stained with Hoechst, and infected cells (magenta) were identified by SARS-CoV-2 N protein immunofluorescence detection. (I) Percentage of SARS-COV-2-infected neurons in monocultures at 24 and 48 hpi. (J) Quantification of SARS-CoV-2 infection at 24 hpi in neurons that were treated with PBS (vehicle) or 2 μg/mL (low), 5 μg/mL (medium), or 20 μg/mL (high) anti-ACE2 or anti-actinin antibodies 1 h prior to infection. *N* = 6 wells per group. Significance is shown in comparison to the vehicle group. All scale bars are 100 μm. Columns and bars represent means ± SEM. Data were analyzed with an ordinary one-way ANOVA followed by Dunnett’s multiple-comparison test (C), an ordinary one-way ANOVA (D and E), or an ordinary one-way ANOVA followed by Tukey’s multiple-comparison test (J). *, *P* < 0.05; **, *P* < 0.005; ***, *P* < 0.0005.

To assess susceptibility of iPSC-derived human neural cultures to SARS-CoV-2, we infected 30- to 32-day-old neuron-astrocyte cocultures with the ancestral SARS-CoV-2 Wuhan strain and analyzed samples by immunofluorescence analysis of viral protein expression at various time points. A representative image of an infected well at 48 h postinfection (hpi) stained with DNA dye Hoechst 33342 (nuclear marker), MAP2, and SARS-CoV-2 nucleocapsid protein (N) is shown in Fig. 2A, with an enlarged area shown in Fig. 2B. The viral N protein was detected both in the neuronal cell body and throughout neurites (dendrites) (Fig. 2B). Additionally, immunolabeling against viral N and double stranded RNA (dsRNA) confirmed the presence of both viral protein synthesis and viral RNA replication in the infected cells (Fig. 2C).

Following the infection at a virus concentration equivalent to a multiplicity of infection (MOI) of 1.5 in Vero E6-TMPRSS2 cells, we found a low level of neuronal infection (around 0.05%) at all analyzed time points (24, 48, and 120 hpi) (Fig. 2D). The level of the neuronal SARS-CoV-2 infection did not differ significantly between the analyzed time points (Fig. 2D), which was in line with previously published work (13). Based on the distribution of the viral N staining at 24 hpi, the infection was mostly localized in the neuronal soma, with proximal neurites only beginning to display signs of N-positive staining (Fig. 2E, arrow). At 48 hpi, it was common to see neurons with both soma and neurites positive for SARS-CoV-2 N (Fig. 2E, arrow). At 120 hpi, all the neurons that were found positive for N had their neurites retracted (Fig. 2E).

To assess whether the infected neurons released infectious virions, we carried out qRT-PCR analysis of the conditioned medium collected from the cells at 0, 24, 48, and 120 hpi. We observed that the levels of viral genome released into the medium increased over time, with the maximum load detected at 48 hpi (Fig. 2F). To assess whether the released viral RNA corresponded to infectious virions, we transferred conditioned medium from infected neuron-astrocyte cocultures to Vero E6 cells, which are highly susceptible to SARS-CoV-2 infection (Fig. 2G and H). As a comparison, we infected Vero E6 cells with different dilutions of the original SARS-CoV-2 stock produced in Vero E6-TMPRSS2 cells (Fig. 2G and H). The presence of infectious virions in the medium tended to increase from 0 to 24 hpi but did not reach the level of statistical significance, likely due to a very low number of infected neurons in our coculture model. At 48 hpi, infectious viruses were no longer detected in the media of infected neuron-astrocyte cocultures, probably due to virus-induced neuronal cell death (Fig. 2G). More studies will be required to accurately determine the extent and kinetics of infectious virus release from infected neurons.

In the neuron-astrocyte cocultures, all of the N-positive cells demonstrated robust staining for the neuron-specific marker MAP2, which suggests that all of the infected cells were neurons. We did not find any infected astrocytes (defined as MAP2-negative cells) in our experiments. Although our data were consistent with previous findings where neurons, but not astrocytes, were susceptible to SARS-CoV-2 infection in human iPSC-derived cultures (10, 12, 13, 15), SARS-CoV-2 infection has been previously observed in human primary astrocytes (27) and iPSC-derived astrocytes (14, 16) in some studies. Some differences in cell culture protocols, such as the length of differentiation, pH, or the presence or absence of fetal bovine serum in the medium might explain the differences in iPSC-derived astrocyte infectibility by SARS-CoV-2. More studies need to be conducted to understand the effects of different culture conditions on viral entry into astrocytes.

Since previous work by Wang et al. demonstrated that the presence of astrocytes exacerbates neuronal susceptibility to SARS-CoV-2 infection in human iPSC neuronal cells (14), we challenged iPSC-derived neuronal monocultures with a dose of SARS-CoV-2 similar to what we used to infect neuron-astrocyte cocultures. The level of infection in neuronal monocultures was comparable to the level of infection in neuron-astrocyte cocultures, which suggested that astrocytes did not facilitate neuronal SARS-CoV-2 infection in our cocultures (Fig. 2I).

### SARS-CoV-2 infection of iPSC-derived neurons is ACE2 dependent.

ACE2 is the primary receptor used by SARS-CoV-2 to enter cells (28). Some of the earliest studies on SARS-CoV-2 challenged the possibility that SARS-CoV-2 could infect brain cells due to the low *ACE2* mRNA levels found in the human brain (29, 30). Since then, some studies have reported ACE2 protein and RNA expression in some human neurons (7, 31, 32), with Song and coworkers further reporting that application of anti-ACE2 antibody prior to infection blocked SARS-CoV-2 in human brain organoids (7). To test whether SARS-CoV-2 infection of human iPSC-derived neurons is dependent on ACE2, we treated the cells with different concentrations of a polyclonal anti-ACE2 antibody (2, 5, or 20 μg/mL) 1 h prior to infection with SARS-CoV-2 (MOI equivalent to 1.5 PFU/cell, as determined in Vero E6-TMPRSS2 cells) (Fig. 2J). At 24 hpi, the presence of anti-ACE2 antibody significantly blocked the infection in a dose-dependent manner (Fig. 2J). An antibody against the intracellular protein actinin, which we used as a negative control for potential unspecific antibody-virus interactions, did not significantly inhibit the infection (Fig. 2J). Overall, these data confirmed previous findings that the neuronal SARS-CoV-2 infection is ACE2 dependent, which is similar to SARS-CoV-2 infection in other cell types (33, 34).

### Virus infection is blocked by inhibition of PIK5K but not serine proteases.

**FIG 3:**
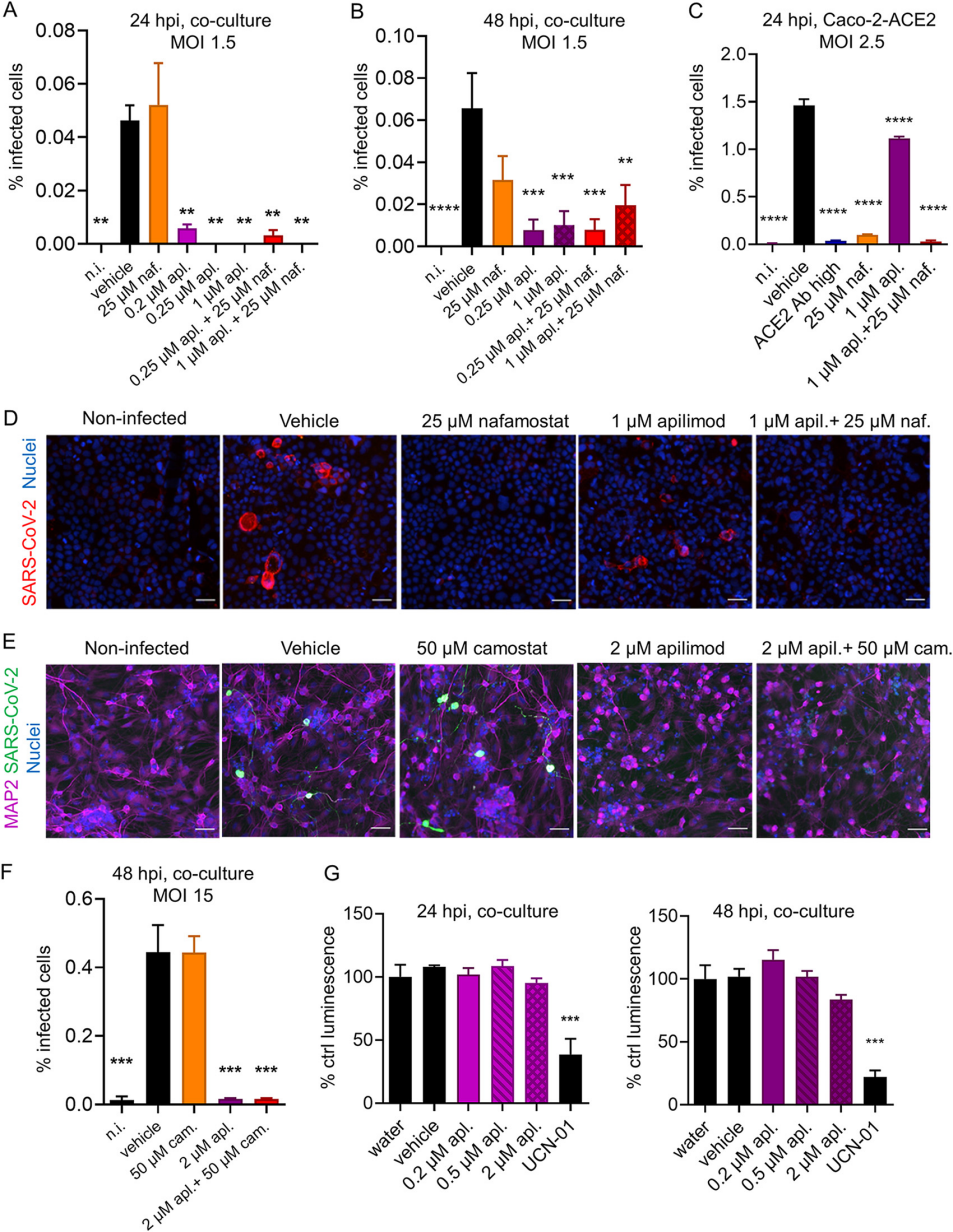
SARS-CoV-2 infection of iPSC-derived neural cultures is blocked by inhibition of PIK5K but not serine proteases. (A and B) Treatment of neuron-astrocyte cocultures with 0.2 to 1 μM apilimod, 25 μM nafamostat, or a combination at 30 min prior to infection with SARS-CoV-2 at an MOI of 1.5. Analysis was at 24 hpi (A) and 48 hpi (B). *N* = 6 samples per group. (C) Treatment of Caco-2-ACE2 cells with 25 μM nafamostat, 1 μM apilimod, a combination, or anti-ACE2 antibody 30 min prior to infection with SARS-CoV-2 at an MOI of 2.5. Analysis was at 24 hpi. *N* = 3 samples per group. (D) Representative immunofluorescence images of SARS-CoV-2 infection (SARS-CoV-2 N protein) in Caco-2-ACE2 cells at 24 hpi at an MOI of 2.5, treated with the indicated drugs. Blue, Hoechst 33342; red, SARS-CoV-2 N. (E) Representative immunofluorescence images of SARS-CoV-2 infection (SARS-CoV-2 N protein) in neuron-astrocyte cocultures at 48 hpi at an MOI of 15, treated with the indicated drugs. Blue, Hoechst 33342; magenta, MAP2; green, SARS-CoV-2 N. (F) Treatment of neuron-astrocyte cocultures with 2 μM apilimod, 50 μM camostat, or a combination of the two drugs, 30 min prior to infection with SARS-CoV-2 at an equivalent MOI of 1.5. Cells were analyzed at 48 hpi after immunofluorescence imaging and image analysis. *N* = 3 wells per group. (G) Cytotoxicity assay in neuron-astrocyte cocultures treated for the indicated times with apilimod at concentrations ranging from 0.2 to 2 μM, or with 9 μM UCN-01. All scale bars are 100 μm. Columns and bars represent means ± SEM, respectively. Data were analyzed by an ordinary one-way ANOVA followed by Dunnett’s multiple-comparison test. *, *P* ≤ 0.05; **, *P* ≤ 0.01; ***, *P* ≤ 0.001; ****, *P* ≤ 0.0001. Significance is shown compared to the vehicle group.

To infect cells, SARS-CoV-2 surface protein spike (S) has to be cleaved by cellular proteases, which is followed by fusion of the virus with the cell membrane or endocytic compartment membranes. Previous studies have reported that SARS-CoV-2 could infect human primary cells through two main pathways: (i) through endocytosis, cathepsin-mediated spike activation, and membrane fusion at late endosomes or lysosomes (35, 36); or (ii) spike activation by TMPRSS2 and direct viral fusion with the plasma membrane or the membrane of early endosomes (22, 23, 37). To investigate the route of infection utilized by SARS-CoV-2 in human iPSC-derived neurons, we used small-molecule inhibitors to block these pathways, alone or in combination. Apilimod blocks PIK5K and therefore disrupts early to late endosomal-lysosomal trafficking, which has previously been shown to block viral infections, such as Ebola virus and SARS-CoV-2 infection of TMPRSS2-negative cells (38–40). When applied 1 h prior to infection at a 0.2 μM concentration, apilimod decreased the number of SARS-CoV-2-infected cells at 24 hpi by more than 80% (Fig. 3A). At 0.25 μM and 1 μM concentrations, it blocked infection completely (Fig. 3A). At 48 hpi, the antiviral effect of the drug was reduced, but infection was still blocked by over 70% (Fig. 3B). Nafamostat, a potent inhibitor of serine proteases, prevents the virus from entering TMPRSS2-expressing cells directly from the plasma membrane or through early endosomes (33). In accordance with the lack of TMPRSS2 expression in our neuron-astrocyte cocultures, nafamostat applied at the saturating 25 μM concentration did not significantly inhibit SARS-CoV-2 infection at either 24 hpi or 48 hpi time points (Fig. 3A and B). A combination of both drug types had an effect similar to that of apilimod alone (Fig. 3A and B), which indicated that serine proteases are not necessary for neuronal infection.

Caco2-ACE2 cells express TMPRSS2 endogenously, which allows direct viral fusion at the plasma membrane (33). Therefore, these cells can serve as a positive control for the inhibitory efficacy of nafamostat. While apilimod had only a small effect on the infection rates in Caco-2-ACE2 cells at 24 hpi, 25 μM nafamostat rendered a >90% decrease in the infection levels (Fig. 3C and D), confirming previous findings that cell surface serine protease inhibitors are capable of blocking SARS-CoV-2 entry in cells where this route is available for the virus. The observed inhibition with nafamostat was similar to that of anti-ACE2 antibody at a 20-μg/mL concentration (high) (Fig. 3C), with both showing almost full inhibition of the infection.

Apilimod inhibited SARS-CoV-2 infection in neurons even when we increased the virus dose 10-fold (Fig. 3E and F). In control-treated cells, this 10-fold increase in virus dose increased the percentage of infected neurons from 0.05 to 0.5%, but the treatment with 2 μM apilimod almost completely blocked the infection (Fig. 3E and F). Notably, apilimod negatively affected the morphology of neurons by causing neurite retraction at 48 h posttreatment (Fig. 3D). Astrocytes were not infected despite the increase in virus dose. Furthermore, we assessed the efficacy of camostat, another serine protease inhibitor that efficiently inhibits SARS-CoV-2 infection in TMPRSS2-expressing cells, with a 50% inhibitory concentration of ~2 μM (41). Camostat did not inhibit SARS-CoV-2 neuron infection, even at a high concentration (50 μM) (Fig. 3E and F), which was consistent with the results obtained with nafamostat (Fig. 3A and B).

Since apilimod induced a clear morphological change in neurons and to make sure that the observed inhibition of virus entry into neurons by apilimod was not due to a general cytotoxic effect, we assessed the potential cytotoxicity of apilimod in neuron-astrocyte cocultures using a luminescence-based CellTiter-Glo 2.0 cell viability assay. 7-Hydroxystaurosporine (UCN-01), which is neurotoxic at high concentrations due to the inhibition of protein kinase C (42), served as a positive control. While UCN-01 was significantly cytotoxic already after 24 h of treatment, apilimod did not show significant cytotoxicity at any of the used concentrations and evaluated time points (Fig. 3G).

**FIG 4:**
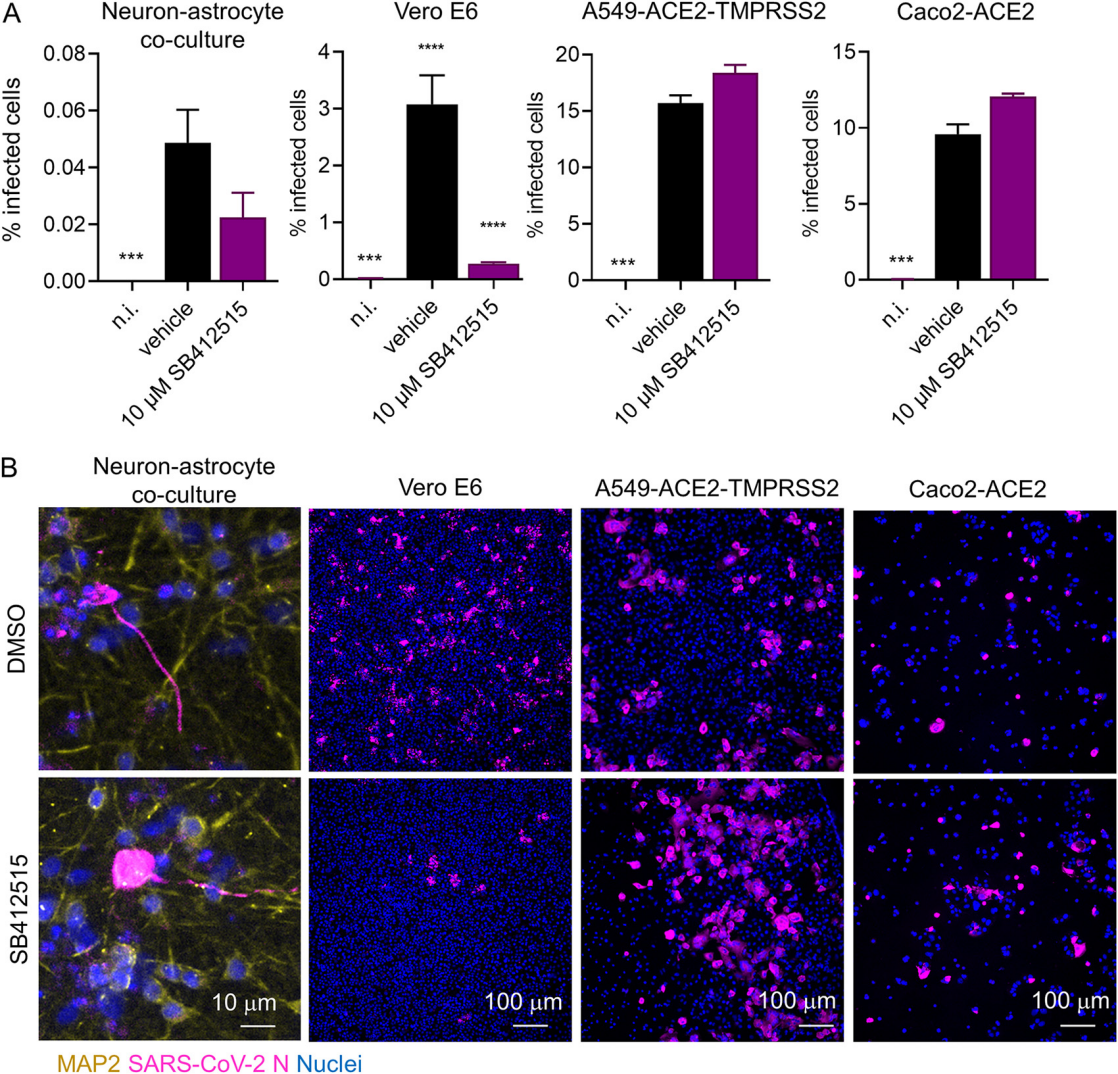
Cathepsin L inhibitor SB412515 does not significantly inhibit SARS-CoV-2 neuron infection. (A) Quantification of SARS-CoV-2 infection in neuron-astrocyte cocultures (48 hpi), Vero E6, A549-ACE2-TMRPSS2, and Caco2-ACE2 cells (24 hpi) treated with 10 μM cathepsin L inhibitor SB412515 starting 30 min before infection. (B) Representative immunofluorescence images of cells infected and treated as described for panel A. Blue, nuclei stained with Hoechst; magenta, infected cells; yellow, neurons (identified by immunofluorescence detection of SARS-VOV-2 N and MAP2 proteins, respectively). Columns and bars represent means ± SEM, respectively. Data were analyzed by an ordinary one-way ANOVA followed by Dunnett’s multiple-comparison test. *, *P* ≤ 0.05; **, *P* ≤ 0.01; ***, *P* ≤ 0.001; ****, *P* ≤ 0.0001. Significance is shown compared to the vehicle group.

Apilimod blocks endosome maturation by inhibiting PIK5K, which prevents endocytic cargoes from being delivered from early to late endosomes and lysosomes. Having established that apilimod blocked SARS-CoV-2 infection in neurons, we tested whether an inhibitor of the lysosomal protease cathepsin L would block neuronal infection. Cathepsin L is known to trigger virus fusion with lysosomal membrane in cells that do not express TMPRSS2. To this end, we treated neuron-astrocyte cocultures with a high concentration (10 μM) of the cathepsin L inhibitor SB412515. Consistent with previous findings (23), SB412515 efficiently blocked infection in Vero E6 cells, which are known to be infected through the endosomal pathway (positive control) (Fig. 4A and B). In contrast, SB412515 was unable to block infection in Caco2-ACE2 and A549-ACE2-TMPRSS2 cells, where the virus enters through a TMPRSS2-mediated pathway (negative controls) (Fig. 4A and B). In neuron-astrocyte cocultures, SB412515 moderately reduced the number of infected neurons in some experiments, but the observed difference was not statistically significant (Fig. 4A and B). Our results indicated that the virus can utilize other cathepsins or lysosomal proteases to infect neurons when cathepsin L is inhibited. For example, cathepsin B can also be involved in SARS-CoV-2 entry (35), and this protein is abundantly expressed in human neurons and neuron-like cells (43).

Since neuronal infection was blocked by an inhibitor of the host factor PIK5K but not by inhibitors of cell surface serine proteases, these data suggested that SARS-CoV-2 infection of iPSC-derived neurons preferentially occurs through the endosomal pathway and not through direct fusion with the plasma membrane following TMPRSS2-mediated cleavage. This could be due to the organ-specific lack of TMPRSS2 and the acidic environment (pH < 6.8) required for direct cell entry at the plasma membrane. For example, the pH of the human nasal mucosa is around 6.6 (23), whereas the brain extracellular fluids have a pH of >7.2 (44). The lack of TMPRSS2 is also known to quickly lead to mutations in the viral spike protein (26), which can select for endosomal-lysosomal cell entry in human neurons that do not endogenously express TMPRSS2.

Drugs aimed at blocking viral infection through the endosomal-lysosomal pathway could potentially be used to prevent or limit neuronal infection by SARS-CoV-2. However, although intravenous administration of apilimod did not show evident adverse effects in human clinical trials, we warn against incautious use of such drugs. In our *in vitro* experiments, apilimod negatively affected the morphology of neurons by causing neurite retraction at 48 h posttreatment (Fig. 3G), even though we did not detect cytotoxicity. Thus, the safety of apilimod should be carefully evaluated in preclinical studies before it can be aimed at treating brain infections.

### Conclusions.

The current study has characterized the SARS-CoV-2 infection pathway in human iPSC-derived cortical-like neurons and has provided evidence that neurons but not astrocytes get infected. The infection relies on ACE2 for entry. When entering the neuronal cells, the virus preferentially uses the endosomal-lysosomal pathway, even though inhibition of cathepsin L did not significantly block infection. In future studies, it will be important to define the identity of the protease(s) involved in virus entry. Viral RNA and low levels infectious viruses were released from infected neurons, but more studies are required to characterize this aspect of the infection.

Nonlethal, low-level infection in the brain might be difficult to trace, but it could still lead to long-lasting negative consequences. Although the infection led to neuronal cell death within 48 to 120 hpi *in vitro*, it is currently unknown how long an infected neuron can survive or release viruses *in vivo*. A deeper understanding of brain infection by SARS-CoV-2 could help us understand if there is a causal connection between direct viral infection of brain cells and the neurological manifestations associated with long COVID. More detailed molecular characterization of the viral entry pathways, mechanisms of assembly, and viral release are needed to develop treatments against COVID-19-associated neurological complications.

## MATERIALS AND METHODS

### Generation and culturing of human iPSCs.

Punch skin biopsies were collected from Finnish healthy males after informed consent. The study has received acceptance from the Research Ethics Committee of the Northern Savo Hospital District (license no. 123.13.02.00/2016).

Skin fibroblasts were expanded in fibroblast culture medium (Iscove’s Dulbecco’s modified Eagle medium [DMEM], 20% fetal bovine serum, 1% penicillin-streptomycin, and 1% nonessential amino acids) as described previously (42) and transduced using a CytoTune iPS 2.0 Sendai reprogramming kit (ThermoFisher Scientific, Waltham, MA, USA) according to the manufacturer’s instructions. Fibroblast culture medium was replaced with Essential 6 medium (E6 supplement; ThermoFisher Scientific) supplemented with 100 ng/mL basic fibroblast growth factor (bFGF; ThermoFisher Scientific) at day 6 postransduction. Reprogrammed iPSC colonies were selected based on morphology at 3 weeks postransduction and expanded on Matrigel (growth factor reduced; Corning, Corning, NY, USA) in Essential 8 medium (ThermoFisher Scientific) at 37°C, 5% CO_2_. Cultures were passaged with 0.5 mM EDTA approximately twice a week. The newly generated human IPSCs were commercially karyotyped (AmbarLab, Barcelona, Spain), and the mRNA expression of pluripotency markers *POU5F* (Oct-4), *NANO*G, and *SOX2* were confirmed using RT-qPCR with the following TaqMan primers (ThermoFisher Scientific): *POU5F1*/Oct-3/4 (Hs00999634_gH), *SOX2* (Hs01053049_s1), and *NANOG* (Hs02387400_g1), as described below. The pluripotency of newly generated iPSCs was verified by immunocytochemistry for stage-specific embryonic antigen 4 (SSEA-4), octamer-binding transcription factor 4 (Oct-4), Nanog, and TRA-1-81 (Fig. S1). All used iPSC lines are listed in Table 1.

### iPSC-derived NGN2 neurons.

iPSC-derived neurons were generated according to a protocol adapted from Nehme et al. (48). Briefly, iPSCs were transduced with a lentivirus containing the NGN2 gene under a tetracycline-inducible promoter (Tet-O-Ngn2-Puro) in combination with lentivirus carrying a FUdeltaGW-rtTA construct (both plasmids from Addgene; lentivirus packing and concentration by Alstem, Richmond, CA, USA) at an MOI of 10 for 1 h. The construct contains a puromycin resistance gene, which allows for selection of neural precursor cells (NPCs). After transduction, the virus was removed, and the cells were cultured normally on 35-mm culture plates coated with Matrigel (growth factor reduced; Merck, Darmstadt, Germany), in E8 medium (ThermoFisher Scientific) plus 50 U/mL penicillin and (50 μg/mL streptomycin). NGN2-transduced iPSCs were expanded under normal iPSC culture conditions, and stock vials were frozen in 10% dimethyl sulfoxide (Sigma, Tokyo, Japan), 10% fetal bovine serum (Biowest, Nuaillé, France) in culture medium.

For neuronal differentiation, a vial of NGN2-transduced iPSCs was thawed and passaged 1 to 3 times under normal culture conditions prior to use. On day 0, neuronal differentiation was initiated by adding 2 μg/mL doxycycline to E8 medium on a 60 to 70% confluent NGN2-iPSC plate. On day 1, medium was switched to N2 medium (DMEM/F12 without l-glutamine, 1% GlutaMAX, 1% N2 [all from ThermoFisher Scientific] and 0.3% glucose) supplemented with 2 μg/mL doxycycline (BioGems, Westlake Village, CA, USA) and dual SMAD inhibitors 0.1 μM LDN-193189 (Sigma), 10 μM SB-431542B (Sigma), and 2 μM Xav939 (BioGems). On day 2, developing NPCs were selected by adding 5 μg/mL puromycin (ThermoFisher Scientific). On day 3, puromycin was removed, dead cells were washed away with base medium, and the cells were returned to N2 medium supplemented with 2 μg/mL doxycycline, 0.1 μM LDN-193189, 10 μM SB-431542B, 2 μM Xav939. On day 4, emerging neurons were plated with or without astrocytes on 0.9- to 13-mm coverslips or glass-bottom 96-well plates coated with 9 to 16 μg/cm^2^ poly-d-lysine and ~1.5 μg/cm^2^ laminin (from a mouse Engelbreth-Holm-Swarm sarcoma; Sigma). Density was 50,000 cells/cm^2^/cell type. Medium was switched to Neurobasal (ThermoFisher Scientific) supplemented with 1% GlutamMAX (ThermoFisher Scientific), 2% B27 without vitamin A (ThermoFisher Scientific), 50 μM nonessential amino acids (ThermoFisher Scientific), 0.3% glucose, and 10 ng/mL glial cell line-derived neurotrophic factor (GDNF), brain-derived neurotrophic factor (BDNF) and ciliary neurotrophic factor (CNTF) (ThermoFisher Scientific). On day 7, proliferation was inhibited with an overnight treatment with 10 μM floxuridine (Bio-Techne, Minneapolis, MN, USA). The cells were maturated for 4 to 6 weeks with 50% medium changes three times a week.

### iPSC-derived astrocytes.

Astrocyte differentiation was initiated by growing confluent iPSC plate in neural maturation medium (Neurobasal; DMEM/F12 without l-glutamine, with 1.7% GlutaMAX, 50 μM nonessential amino acids, 0.5 mM sodium pyruvate, 0.5% N2, 1% B27 with vitamin A, 50 μM beta-mercaptoethanol, 2.5 μg/mL insulin, 50 U/mL penicillin, 50 μg/mL streptomycin) supplemented with dual SMAD inhibitors 10 μM SB-431542B and 200 μM LDN-193189 for 10 to 12 days. Resulting NPCs were split 1:2 by scraping and plating on 1.5-μm/cm^2^ laminin-coated 35-mm cell culture dishes. The NPCs were expanded for 2 to 4 days in NMM supplemented with 20 ng/mL bFGF. Then, the cells were detached and moved to ultralow attachment plates (Corning) in astrodifferentiation medium (DMEM/F12 without l-glutamine, with 1% GlutaMAX, 50 μM nonessential amino acids, 1% N2, 50 U/mL penicillin, 50 μg/mL streptomycin, 0.5 IU/mL heparin) supplemented with 10 ng/mL bFGF and epidermal growth factor. Astrospheres were cultured for 6 months and cut manually when necessary. For experiments, astrospheres were dissociated with StemPro Accutase (ThermoFisher Scientific) for 10 min, triturated into a single-cell suspension, and plated on culture dishes. For characterization and SARS-CoV-2 infection in monoculture, astrocytes were plated on growth factor-reduced Matrigel (Corning) at a density of 50,000 cells/cm^2^ and maturated in astrodifferentiation medium supplemented with 10 ng/mL BMP-4 and CNTF (ThermoFisher Scientific) for 2 to 4 weeks.

### SARS-CoV-2 virus.

SARS-CoV-2 virus (sequence corresponding to the Wuhan strain) was produced in Caco-2 cells (passage 3), and titers were determined by standard plaque assay in Vero E6 cells expressing TMPRSS2. The virus genome was sequenced by deep sequencing at full genome coverage as described elsewhere (26), and the presence of an intact “furin cleavage site” in the spike gene was confirmed (sequence accession number MW718190).

### Virus infections.

Experiments involving infection of cells with SARS-CoV-2 were performed in a biosafety level 3 (BSL-3) facility of the University of Helsinki under all required university permissions. Infection of cells was performed in 96-well plates in neurobasal medium (ThermoFisher Scientific) supplemented with 1% GlutaMAX, 2% B27 without vitamin A, 50 μM nonessential amino acids, 0.3% glucose, and 10 ng/mL GDNF, BDNF, and CNTF. Before application to the cells, SARS-CoV-2 was preincubated in the neurobasal medium at 37°C for 30 min. After the infection, the cells were incubated at 37°C with 5% CO_2_ supplementation for 24, 48, or 120 hpi. For the Caco-2 cell experiment, infection was carried out in DMEM (Sigma) supplemented with 4,500 mg/liter glucose, sodium pyruvate, sodium bicarbonate, 1% l-glutamine, 1% nonessential amino acids, 2% fetal bovine serum, penicillin, and streptomycin. The neuronal infections presented in this work are from four independent batches of iPSC-derived neuron-astrocyte cocultures and corresponding infections, typically two different neuronal lines were used for each round of induction, and 3 to 6 replicate wells were included for each treatment per plate. For each repetition, different aliquots of virus and drug stocks were used.

### Anti-ACE2 and anti-actinin 1 antibody treatment on live cells.

Different concentrations of anti-ACE2 and anti-actinin 1 antibodies (low, 2 μg/mL; medium, 5 μg/mL; high, 20 μg/mL) were added to the cells 30 min prior to the infection with the virus.

### Drug treatment.

The cells were treated with 0.2 μM, 0.25 μM, 1 μM, or 2 μM apilimod dimesylate (Tocris catalog number 7283, batch 1A/257560), 50 μM camostat mesylate (Bio-Techne catalog number 3193, batch 2B/242261), 25 μM nafamostat mesylate (Bio-Techne catalog number 3081, batch 6A/257562), or combinations of these drugs 30 min prior to the infection with the virus. For direct inhibition of cathepsin L, the cells were treated with 10 μM or 20 μM SB412515 30 min prior to infection with the virus.

### Cytotoxicity assay for apilimod.

For the cytotoxicity assay with apilomod, the cells were treated with different doses of apilimod (0.2, 0.5, and 2 μM) for 24 and 48 h. Treatment with 9 μM UCN-01, which is known to induce cell damage at this concentration, was used as a positive control. Cytotoxicity was measured using a CellTiter-Glo 2.0 cell viability assay (Promega, Madison, WI, USA) with the protocol provided on the manufacturer’s website. Briefly, the cells were removed from the incubator and left at room temperature for 30 min. Then, 100 μL of CellTiter-Glo reagent (Promega) was added on top of the 100 μL of cell medium at room temperature. The cells were lysed for 2 min on an orbital shaker and left on the bench for 10 min to stabilize the luminescence signal. Luminescence was measured using a Hidex Sense microplate reader (Hidex, Turku, Finland) with a 1-s integration time.

### Immunocytochemistry for cell type characterization.

Cell cultures were fixed with 4% paraformaldehyde (PFA) and washed twice with phosphate-buffered saline (PBS). Cells were permeabilized for 20 min with 0.25% Triton X-100 in PBS or left unpermeabilized (for SSEA-4 and TRA-1-81). Unspecific binding was blocked with 5% normal goat serum in PBS (blocking buffer). Primary antibodies were diluted in blocking buffer and incubated overnight at 4°C. The next day, the samples were washed three times for 10 min with PBS. Secondary antibodies were diluted 1:1,000 in blocking buffer and incubated for 1 h at room temperature. Samples were washed three times for 10 min with PBS and stained with the nuclear marker 4′,6-diamidino-2-phenylindole prior to mounting with Fluoromount (ThermoFisher Scientific). Characterization was done for six neuronal cell lines and four astrocyte cell lines. ACE2 protein expression in neuron-astrocyte cocultures was assessed using cocultures derived from one iPSC cell line (MADGIC 8cl1). Four coculture wells were analyzed for ACE2 expression and compared to similarly stained Caco2-ACE2 (positive control) and A549 (negative control) cells.

### Immunohistochemistry for virus-infected samples.

The cells were fixed with 4% PFA in PBS at room temperature for 20 min. PFA was removed, and the cells were incubated in PBS at 4°C until the staining was performed. The virus was inactivated with UV radiation (5,000 J/m^2^ dose) before removal of the samples from the BSL-3 laboratory. Before permeabilization, the cells were incubated in 50 mM ammonium chloride (NH_4_Cl) in PBS to quench free aldehyde groups remaining postfixation at room temperature for 20 min. Then, the cells were permeabilized with 0.1% Triton-X in PBS, and the nuclei were stained with 1:1,000 Hoechst 33342 in Dulbecco medium containing 0.2% bovine serum albumin (BSA-DPBS) for 10 min. The cells were washed once with 0.2% BSA-DPBS and incubated in primary antibody at 4°C overnight. On the following day, the cells were washed twice with 0.2% BSA-DPBS and incubated in fluorescent dye-conjugated secondary antibodies for 1 h at room temperature. After that, the cells were washed with 0.2% BSA-DPBS three times, and 100 μL of PBS per well was added.

### Antibodies.

A full list of primary and secondary antibodies used in the study is provided in Table 2.

**TABLE 2:** Antibodies used in the study

Antibody	Concn used	Manufacturer	Reference or catalog no.
ACE2 goat	1:1,000	Bio-Techne	AF933
Actinin goat	2, 5, 20 μg/mL	Bio-Techne	AF8279
CTIP2 rat	1:500	Abcam	ab18465
Cux1 mouse	1:500	Abcam	ab54583
GABA rabbit	1:500	Sigma	A2052
GFAP rabbit	1:500	Agilent	Z033429-2
MAP2 chicken	1:1,000	Abcam	ab92434
Nanog	1:100	ThermoFisher Scientific	MA1-017
Nucleocapsid rabbit	1:2,000	Jussi Hepoioki, University of Helsinki	51
Oct4	1:400	Merck	MAB4401
S100β rabbit	1:500	Abcam	ab52642
SSEA4	1:400	Merck	MAB4304
TRA-181	1:200	Merck	MAB4381
Tubulin-3 mouse	1:2,000	PerkinElmer	801201
Vglut1 rabbit	1:500	Sigma	V0389
Goat anti-mouse 488	1:400	ThermoFisher Scientific	A11001
Goat anti-mouse Alexa 633	1:500	ThermoFisher Scientific	A21052
Goat anti-rabbit Alexa 488	1:1,000	ThermoFisher Scientific	A11008
Goat anti-chicken Alexa 568	1:1,000	ThermoFisher Scientific	AB_2534098
Chicken anti-goat Alexa 488	1:1,000	ThermoFisher Scientific	A21467
Donkey anti-rabbit Alexa 647	1:1,000	ThermoFisher Scientific	A31573
Donkey anti-mouse Alexa 555	1:1,000	ThermoFisher Scientific	A32773

### High-throughput imaging to detect the virus.

High-throughput imaging was carried out using an ImageXpress Nano microscope (Molecular Devices) at the Light Microscopy Unit of the University of Helsinki. We used two Nikon objectives: 10× 0.3-numerical aperture (NA) Plan Fluor, WD 16 mm (pixel size, 0.655 μm) and 20× 0.45-NA S Plan Fluor ELWD, WD 8.2 to 6.9 mm (pixel size, 0.328 μm). Detailed information on the filter specifications for different channels can be found at https://wiki.helsinki.fi/display/LMU/MolecularDevices+Nano.

### Image analysis of high-throughput images.

High-throughput image analysis was carried out using Cell Profiler 4 (49). Approximately 10,000 cells per sample were analyzed. First, the nuclei were identified on the image channel dyed with Hoechst 33342 by providing a typical diameter of the objects using the Otsu thresholding method. Then, the objects identified as nuclei were expanded by a few pixels to approximately represent the borders of a cell. The channel with the MAP2 staining was overlaid with the expanded nuclei, and a threshold for an average signal intensity of MAP2-positive cells was chosen. Cells displaying MAP2 intensity above the chosen threshold were classified as neurons, and other cells were classified as astrocytes. Both classes of cells were overlaid with an image channel where staining for the virus N-protein was carried out. Cell bodies demonstrating intensity of staining above a defined threshold were counted as virus-positive cells. The percentage of cells positive for the virus N protein from the total population of cells of their cell type was plotted. For the control experiment in Caco-2 cells, the pipeline was similar, but no cell type-specific markers were used, as the cell line was homogeneous.

### Statistical analysis of high-throughput images.

Statistical analysis of the data was performed in GraphPad Prism 6. A ROUT test was performed before the analysis to identify outliers. A two-tailed unpaired Student's *t* test was performed when two conditions were compared. One-way analysis of variance (ANOVA) was used when more than two groups were analyzed. When the main effect was found to be statistically significant, *post hoc* multiple-comparisons tests were carried out. Differences were considered statistically significant when *P* was <0.05. Data in figures are presented as means ± standard errors of the means (SEM). Significance is shown in comparison to the vehicle group (or as an exception, to the lowest time postinfection for Fig. 2D, F, G, and I).

### qRT-PCR to assess RNA expression of iPSC, astrocyte, and cell surface markers.

Levels of ACE2, GFAP, S100β, NRP1, and TMPRSS2 in our iPSC-derived neurons, astrocytes, and NPCs were assessed with qRT-PCR. First, RNA was isolated from cultured neurons, astrocytes, and control cell lines Caco2, Caco2-ACE2, and A549 using an RNeasy minikit (Qiagen, Hilden, Germany) following the manufacturer’s instructions. The concentration of RNA was measured using a NanoDrop system, and 500 ng of RNA was converted into cDNA. First, 500 ng of RNA was diluted in water and mixed with random hexamer primer (ThermoFisher Scientific). The samples were incubated 5 min at 65°C in a C1000 thermal cycler (Bio-Rad, Hercules, CA, USA). Then, a synthesis mixture (10 mM deoxynucleoside triphosphates, RNase inhibitor, and Maxima reverse transcriptase in reaction buffer; ThermoFisher Scientific) was added to the samples and cDNA synthesis was run for 30 min at 50°C. Quantitative RT-PCR was run using a Maxima probe/ROX qPCR master mix and the following TaqMan primers: *ACE2* (HS01085333_m1), *GAPDH* (Hs99999905_m1), *GFAP* (Hs00909233_m1), *Neuropilin-1* (Hs00826128_m1), *S100β* (Hs00902901_m1), and *TMPRSS2* (HS01122322_m1) (ThermoFisher Scientific) on a Bio-Rad CFX96 real-time system. The samples were run at 95°C for 10 min followed by 40 cycles of 95°C for 15 s, 60°C for 30 s, and 72°C for 30 s. The results were normalized to human *GAPDH* expression using the Q-gene program (50). Receptor RNA expression in neurons and astrocytes was compared to positive control lines Caco2 and Caco2-ACE2, which overexpress ACE2, and the negative control cell line A549, which is known to not express ACE2 or TMPRSS2. Individual variations between iPSC lines and brain cells derived from different iPSC lines were assessed by repeating qRT-PCR for 2 to 4 cell lines each. The specificity of the primers was confirmed by running 10 μL of the PCR products in a 2% agarose gel containing Gel Red (Biotium catalog number 41003) at a final concentration of 0.5 μg/mL, followed by electrophoresis at 90 V for 30 min. The gel was exposed to UV light and imaged with a Bio-Rad imaging system (Gel Doc 2000). The expected size of the respective DNA amplicons was determined by comparison to a DNA size marker (Thermo Scientific catalog number SM1333).

### qRT-PCR to assess viral release.

SARS-CoV-2 RNA was harvested from the cell medium at various time points postinfection and stored in viral lysis buffer with RNA supplements (50 μL of sample medium in 560 μL of AVL buffer supplemented with 1% carrier RNA; Qiagen). RNA was extracted using a QIAamp viral RNA minikit (Qiagen). RNA concentration and quality were evaluated using a NanoDrop 2000 spectrophotometer (ThermoFisher Scientific). qRT-PCR was run in triplicates using reverse transcriptase-negative and template-negative controls and TaqMan Fast Virus 1-step MasterMix (ThermoFisher Scientific) (5 μL of the sample in a 20-μL total volume). Primers and probe used for the reaction were ordered from Metabion: RdRP-SARSr-F2, 5′-GTG ARA TGG TCA TGT GTG GCG G-3′ as the forward primer; RdRP-SARSr-R2, 5′-CAR ATG TTA AAS ACA CTA TTA GCA TA-3 as the reverse primer; RdRP-SARSr-P2, 5′–6-carboxyfluorescein-CAG GTG GAA CCT CAT CAG GAG ATG C–BHQ-1–3′ as the probe. The qRT-PCR was run with AHDiagnostics Agilent Technologies Stratagene Mx3005P using the following steps: reverse transcription for 5 min at 50°C, initial denaturation for 20 s at 95°C, and two amplification steps at 95°C for 3 s and 60°C for 30 s (the amplifications steps were repeated for 40 cycles).

### Microelectrode array.

Electric activity of neuron astrocyte-cocultures was assessed using the Maestro Edge multiwell microelectrode array system (Axion). The cells were plated on 24-well Cytoview MEA plates (Axion) at a density 60,000 neurons + 60,000 astrocytes per well and cultured for 3 weeks prior to starting the recordings. Each well contained 16 electrodes per well with 50-μm electrode diameter and 350-μm electrode spacing. The activity was recorded at 37°C, 5% CO_2_ for 10 min until day 35. The signal was sampled at a frequency of 12.5 Hz and filtered with a digital low-pass filter 3-kHz Kaiser Window and digital high-pass filter 200-Hz IIR. The noise threshold was set at 5 standard deviations. Bursts were detected with the following interspike interval threshold settings: minimum number of spikes, 10; maximum interspike interval, 100 ms. Network bursts were detected with the following settings: minimum number of spikes, 90; maximum interspike interval, 20 ms; minimum percentage of participating electrodes, 33%. Characterization was done using five cell lines, and all values were calculated as a mean of three wells.

### Data availability.

Further information on methodologies and data can be provided upon request.
