# Supplementary material for: SARS-CoV-2 Infection of Human Neurons Is TMPRSS2 Independent, Requires Endosomal Cell Entry, and Can Be Blocked by Inhibitors of Host Phosphoinositol-5 Kinase
Source: J Virol. 2023 Apr 11;97(4):e00144-23. doi: 10.1128/jvi.00144-23 (PMC10134833; doi:10.1128/jvi.00144-23)
Supplement: Supplemental file 1 — Fig. S1 and S2. Download jvi.00144-23-s0001.pdf, PDF file, 0.5 MB [file jvi.00144-23-s0001.pdf]

1 **Supplementary figures:**

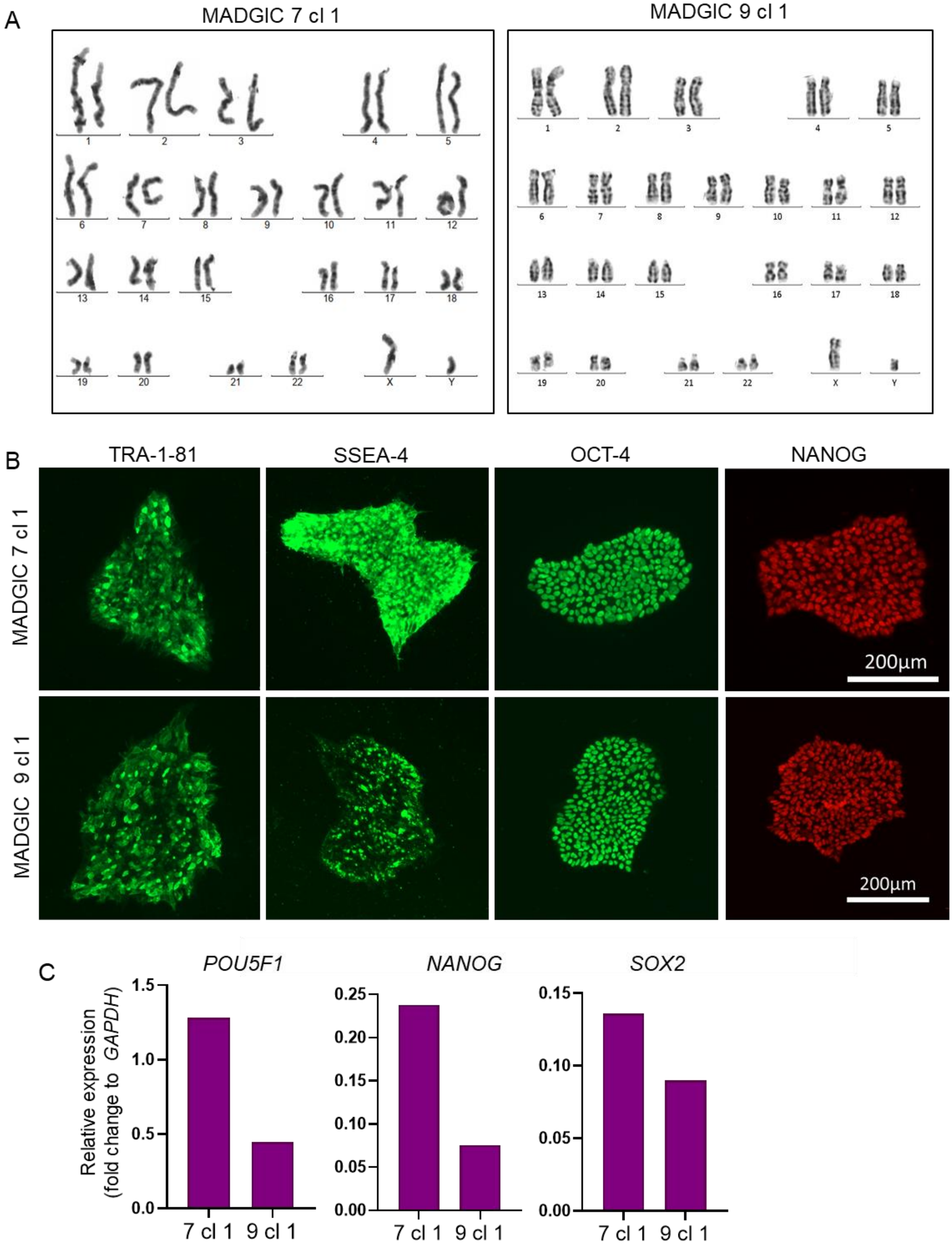

2

Supplementary Fig. 1. Characterization of previously unpublished iPS cell lines.

A. Karyotypes of MADGIC 7 cl1 and 9 cl 1 iPS cell lines.

B. Immunocytochemical staining (MADGIC 7 cl 1 & MADGIC 9 cl 1) with TRA-1-81, stage-specific embryonic antigen 4 (SSEA-4), octamer-binding transcription factor 4 (OCT4) and NANOG antibodies. Scale bar 200 μm.

C. Mean normalized mRNA expression of pluripotency markers POUF5/OCT-4, NANOG and SOX2. Normalized to human GAPDH expression.

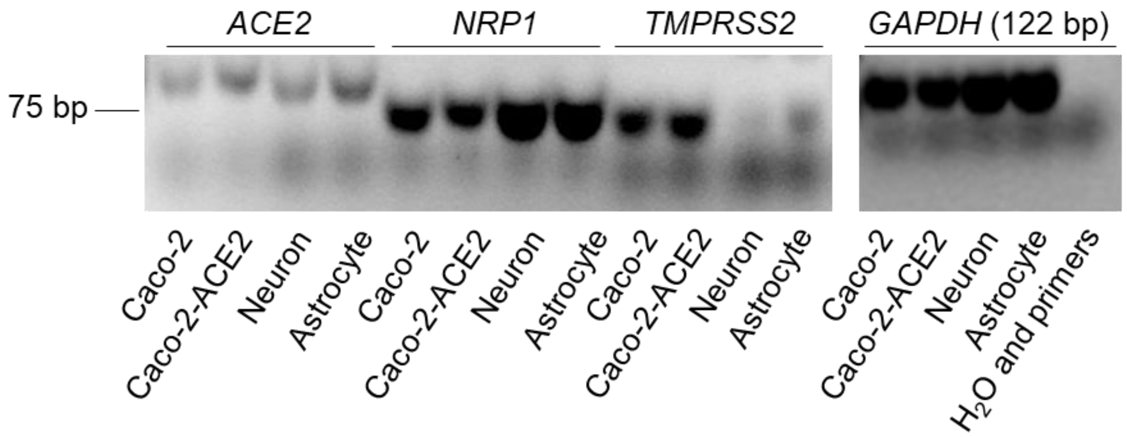

Supplementary Fig. 2. Agarose gel electrophoresis of *ACE2*, *NRP1* and *TMPRSS2* primers (TaqMan) in indicated cell types. The specificity of the primers was confirmed by running an equal volume of the PCR products in 2% agarose gel containing Gel Red followed by electrophoresis. The expected size of the respective DNA amplicons was determined by comparison to a DNA size marker.
